# Stigma and discrimination against adolescents living with perinatal HIV in Thailand: caregivers' perceptions

**DOI:** 10.3389/fpubh.2025.1535004

**Published:** 2025-03-05

**Authors:** Audrey Geoffroy, Wasna Sirirungsi, Parinya Jongpaijitsakul, Wanna Chamjamrat, Chutima Ruklao, Manoosin Kongka, Usa Sukhaphan, Somporn Sathan, Sriphan Thina, Tassawan Khayanchoomnoom, Sophie Le Coeur, The TEEWA Study Group

**Affiliations:** ^1^French National Institute of Population Studies (INED), Aubervilliers, France; ^2^Faculty of Associated Medical Sciences, Chiang Mai University, Chiang Mai, Thailand; ^3^Associated Medical Sciences/PHPT Research Collaboration, Chiang Mai, Thailand; ^4^Prapokklao Hospital, Chantaburi, Thailand; ^5^Phayao Hospital, Phayao, Thailand; ^6^Sanpatong Hospital, Chiang Mai, Thailand; ^7^Hat Yai Hospital, Hat Yai, Thailand; ^8^Chiang Kham Hospital, Chiang Kham, Phayao, Thailand; ^9^Doi Saket Hospital, Chiang Mai, Thailand; ^10^Mahasarakam Hospital, Mahasarakam, Thailand

**Keywords:** perinatal HIV, stigmatization, discrimination, Thailand, bullying, adolescent

## Abstract

**Background:**

Thailand has been particularly affected by the HIV epidemic in the middle of the years 1990s. Thousands of children living with perinatal HIV have been exposed to HIV-related stigma/discrimination, but its frequency and expressions have been little studied. Our objectives were to assess, among adolescents living with perinatal HIV, the prevalence of stigma/discrimination, the factors associated with it and its expressions.

**Methods:**

All caregivers of adolescents aged 12–19 years living with perinatal HIV and receiving antiretrovirals in 20 hospitals throughout Thailand were invited to complete a face-to-face questionnaire on their adolescent's life, and to report their adolescents' experiences of stigma/discrimination. Stigma/discrimination as perceived by the caregivers was analyzed using both quantitative and qualitative approaches.

**Results:**

A total of 712 adolescents living with perinatal HIV and their caregivers were interviewed as part of the TEEWA study between March 2010 and November 2012. Of the 572 adolescents living in family settings, 464 had their HIV-status known in the community. Among them, the overall stigma/discrimination prevalence was 46%. The multivariable analysis showed that the risk of being stigmatized was nearly 3 times higher in the northeast region (OR: 2.93, 95%CI: 1.36–6.45) and when having a low intellectual ability (OR: 3.35, 95%CI: 1.66–7.10). It was nearly twice higher in case of conflicts with caregivers (OR: 1.81, 95%CI: 1.17–2.79) and when caregivers were members of a support group (OR: 2.28, 95%CI: 1.48–3.53), while having a BMI >18.5 was associated with a lower risk of stigma/discrimination (OR: 0.61, 95%CI: 0.37–0.98). Expressions of stigma/discrimination included bullying, social isolation, behavioral discrimination and public disclosure. Consequences of stigma/discriminations included voluntary withdrawal from school, painful awareness of HIV status, marginalization from the community, and separation of drinks and food.

**Conclusion:**

We found that the prevalence of stigma/discrimination among adolescents living with perinatal HIV was high. Despite existing policies, stigma eradication remains necessary to normalize their life as they grow into adulthood and may face the consequences of past/current discrimination in terms of access to university studies or occupation, at work, in the community or in their romantic life.

## 1 Introduction

Recognized by the United Nations General Assembly in 2006 as “a critical element in combating the global HIV/AIDS pandemic” (1), HIV-related stigma has been defined as negative beliefs, feelings and attitudes toward people living with HIV, groups associated with people living with HIV and other key populations at high risk of HIV infection (2). In addition, UNAIDS has characterized discrimination as the unfair and unjust action taken against an individual or group because of their real or perceived status or attributes, such as a health condition (e.g. HIV), socioeconomic status, gender, race, sexual identity or age (3). HIV-related stigma/discrimination is recognized as an important cause of inequality (4), leading to poorer resources, socio-emotional skills and access to prevention and healthcare (5). However, the extent of stigma/discrimination is difficult to assess because people may keep their HIV status secret for fear of being stigmatized or discriminated against.

Stigma/discriminations in perinatally infected children and adolescents has multiple characteristics. Among them, orphanhood, in affected communities, might be interpreted as the loss of parents because of AIDS and the HIV-stigma toward parents can be passed on to their children (6, 7). HIV-stigma could impede an adolescent's education and cognitive development (8, 9). A lower intellectual capacity could be a cause of stigmatization in itself and reinforce HIV-stigma (10). Poor health has been found to be a cause of stigma (4), notably when associated with visible symptoms of HIV, like lipodystrophy (11–13). Fear of stigma or discrimination can lead to poor access to HIV diagnosis (14) and reduced adherence to antiretroviral treatment, disease progression, health problems and, in turn, stigma (15). Adolescents might have a lower perception of their happiness (6, 16), and on the other hand, those who do not know their HIV status may not perceive any social isolation (17, 18). As for caregivers, their participation in support groups would enable them to be better informed about HIV and to be more sensitive to stigma and discrimination (19). Finally, the poor financial situation of the caregivers may reinforce stigma (6, 20, 21). Food accessibility has been specifically mentioned as a priority need for adolescents' caregivers, as good nutritional status would reduce the visibility of the disease (22).

Thailand was particularly affected by the HIV epidemic from the late 1980s onwards. At first, the HIV epidemic impacted intravenous drug users, then sex workers, their clients and eventually their non-sex workers girlfriends or wives, confirming the generalization of the epidemic (23). According to the Thai Ministry of Public health, in the early 2010s there were more than 12,000 HIV-infected adolescents aged 12–19 years old in the country, of whom at least 9,000 were receiving antiretroviral therapy (24). In addition, according to UNAIDS, there were ~520,000 people living with HIV in Thailand in 2021. Of these, around 2,000 were under 15 years of age (25), a dramatic reduction reflecting the success of the national Prevention of mother-to-child transmission (PMTCT) program (26).

While there is literature on HIV-related stigma in adults in Thailand (21, 27–30), little is known about the specific population of children and adolescents living with perinatally-infected HIV. To our knowledge, only one survey conducted at Chiang Mai University Hospital in northern Thailand has assessed stigma from the perspectives of caregivers (31) and adolescents (32). It shows that discriminatory attitudes were most prevalent among caregivers themselves, and that HIV-related stigma/discrimination was identified in all aspects of adolescents' lives.

To better understand the circumstances and expressions of stigma among children and adolescents living with perinatal HIV, we analyzed data from a multicenter cross-sectional study conducted among adolescents and their caregivers in Thailand (33). The aim of this analysis was to assess the prevalence of stigma/discriminations and its associated factors among children/adolescents living with perinatal HIV in Thailand, as perceived by their caregivers. We also conducted a qualitative description of the experiences of stigma/discrimination during childhood or adolescence as perceived by their caregivers.

## 2 Materials and methods

We used the quantitative and qualitative information obtained from caregivers of adolescents living with perinatal HIV who participated in the Teens Living With Antiretrovirals (TEEWA) study (33). Briefly, the TEEWA study is a cross-sectional study conducted between January 2010 and November 2012 to examine the living conditions of adolescents living with perinatal HIV in Thailand compared with a control group from the general population. Caregivers (or legal guardians) of adolescents aged 12–19 years who were receiving antiretrovirals (ART) in 20 public hospitals across Thailand were invited to participate in the study with their adolescent child. At the hospital, the adolescents completed a detailed self-administered questionnaire about their daily lives. Because some adolescents did not know their HIV status, the adolescent questionnaire did not include questions about HIV. In a face-to-face interview conducted in the hospital, caregivers were asked about the adolescent's sociodemographic status, life and medical history, including HIV diagnosis and treatment history, experiences of stigma/discrimination, caregivers' perception of their adolescent's wellbeing, and their relationship. Clinical, virological and immunological information was extracted from medical records by the attending hospital nurse. Written informed consent and assent was obtained from caregivers and adolescents, respectively. Details regarding the TEEWA study have been published elsewhere (33).

The study was approved by the Faculty of Associated Medical Sciences of Chiang Mai University (ref: AF02-014) and by the ethics committees of the participating hospitals. All data were pseudo-anonymized using unique identifiers. Data is available upon request.

### 2.1 Inclusion criteria

Only adolescents living in a family environment were included, as the question on stigma/discrimination was not asked for those living in orphanages. In fact, they usually live in a separate environment where they have limited interaction with the community. Moreover, these children/adolescents may not talk to orphanage staff about their experiences of stigma/discrimination. In addition, adolescents whose HIV status was not known from the community (as reported by the caregivers) were excluded from the analysis. In fact, when the HIV status was not disclosed or was kept secret from the community, adolescents were not exposed to HIV-related stigma and discrimination. Community (≪ชุมชน≫ in Thai language) includes all people present in the adolescent's environment apart from family members.

### 2.2 Variables of interest: stigma or discrimination

Experiences of stigma/discrimination were recorded by asking caregivers whether they knew that the adolescent in their care had ever experienced stigma or social discrimination because of their HIV status. The settings in which these experiences of stigma or discrimination had occurred were also recorded: family, friends, school, hospital, in the community. Within each setting, the frequency of occurrence was also reported.

### 2.3 Quantitative analysis

#### 2.3.1 Covariates

The TEEWA questionnaire was built to assess the living situation of adolescents living with perinatal HIV, but not specifically to address the question of stigma. Characteristics obtained from the caregiver interviews included the adolescent's gender; age; region of residence (center, north, northeast, or south); orphan status, classified as at least one parent alive vs. both parents known to be dead; school delay (i.e. having repeated a grade); caregiver's perceived intellectual ability of the adolescent (good/very good, fair/low/very low); perceived adolescent's health (very good/good/fair/poor/very poor); perceived happiness (very happy/happy/fair/unhappy/very unhappy); presence of conflicts with the adolescent; caregiver's knowledge of the adolescent's awareness of his/her HIV status (yes, no or unsure); age at ART initiation; adherence to ART (very good/good/fair/poor/very poor).

Clinical characteristics of adolescents obtained from medical records included the adolescent's most recent height and weight, CD4 cell count, and HIV-1 RNA viral load. The body mass index (BMI) was calculated, and underweight was defined as < 18.5 kg/m^2^.

Information about the adolescent's caregiver included gender; age; relationship with the adolescent (parent, grandparent or sibling, aunt or uncle, and other); perceived financial situation (very good/good/fair/difficult/very difficult); perceived current health status (very good/good/fair/poor/very poor); and membership in a support group for people living with HIV/AIDS.

#### 2.3.2 Statistical analyses

We first describe the characteristics of adolescents living in family setting overall and provide the percentages of adolescents whose HIV-status is known in the community. We then provide the percentages of adolescents reporting stigma/discrimination among those whose HIV status was known in the community. The chi-square test was used to assess differences between groups, with statistical significance set at *p* < 0.05.

Logistic regression was used to identify factors associated with any experience of discrimination and was carried out in two stages: Covariates with statistical significance < 0.15 in the univariable analysis were included in the multivariable analysis (34). Two additional logistic regressions were performed to assess the factors associated with “repeated stigma/discrimination,” i.e. when it was reported to occur regularly in at least one circumstance, and “diverse stigma/discrimination,” i.e. when it was reported in at least two circumstances. Statistical analyses were performed using R software (version 4.1.2).

### 2.4 Qualitative analysis

Caregivers were also asked to describe their adolescents' experiences of stigma by answering the following question: “Is there any particular experience of stigma/discrimination concerning your child that you can tell us about.” All verbatims were recorded and translated into English by the interviewers. They were all included in the analysis. Inductive analysis was used to identify the main themes and was checked by two of the authors. Comments were classified according to these themes and their frequency were reported. The relationship between the caregiver and the child was specified when reporting verbatims. Each selected comment in the results section came from a different family.

## 3 Results

### 3.1 Characteristics of the study population

**Figure 1:**
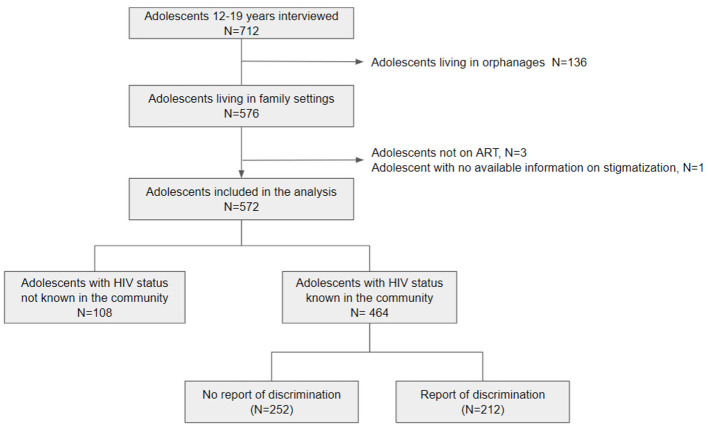
Flow chart describing the selection of the study population for the analysis.

A total of 712 adolescents living with perinatal HIV and their caregivers were interviewed as part of the TEEWA study between March 2010 and November 2012. Among the adolescents, those living in orphanages (*n* = 136), those not currently receiving ART (*n* = 3) and one with missing information on stigma were excluded, leaving a total of 572 adolescents. Figure 1 presents the study population selection.

Table 1 provides a description of the adolescents' and their caregivers' characteristics obtained from the caregivers' questionnaire. Fifty eight percent were female. Their median age was 14 years. The majority were living in the North. HIV status was more often known in the community in the North and North-East regions. Moreover, 43% were orphan from both parents, 88% were aware of their HIV status, 67% had a CD4 count >500 cells/mm^3^, and 82% had a viral load below 50 copies/ml.

**Table 1:** Characteristics of adolescents living in family setting overall, percentages of adolescent's whose HIV status is known in the community, and percentages of adolescents with stigma/discrimination experiences as reported by their caregivers.

	**Overall adolescents living in family setting**	**Adolescents whose HIV status was known in the community**	*p* ^*^	**Adolescents with stigma/discrimination experiences reported by their caregivers**	*p* ^**^
	***N*** **(% of category)**	***N*** **(% status known)**		***N*** **(% with stigma)**	
**Characteristics of the adolescents**					
Total	572 (100)	464 (81)		212 (46)	
**Gender**			0.70		0.70
Female	334 (58)	269 (80)		121 (45)	
Male	238 (42)	195 (82)		91 (47)	
Age (years)	14.4	14.5	0.40	14.5	0.80
Median [IQR]	[13.1–16.0]	[13.2 16.0]		[13.3–15.9]	
12–13	203 (35)	159 (78)		74 (46)	
14–15	195 (34)	159 (82)		75 (47)	
16–19	174 (30)	146 (84)		63 (43)	
**Region**			< 0.01		< 0.01
Center	139 (24)	83 (60)		31 (37)	
North	336 (59)	315 (94)		142 (45)	
North-East	66 (12)	54 (82)		33 (61)	
South	31 (5)	12 (39)		6 (50)	
**Orphan from both parents** ^***^			< 0.01		0.30
Yes	245 (43)	220 (90)		95 (43)	
No	327 (57)	244 (75)		117 (48)	
**School delay (repeated a school year)**			0.10		0.40
Yes	87 (15)	76 (87)		38 (50)	
No	485 (85)	388 (80)		174 (45)	
**Perception of adolescent's intellectual capacity**			0.10		< 0.01
Low	82 (15)	72 (88)		47 (65)	
Normal	490 (85)	392 (80)		165 (42)	
**Perception of adolescent's health**			0.40		0.60
Good or very good	445 (78)	357 (80)		161 (45)	
Less than good	127 (22)	107 (84)		51 (48)	
**Perception of adolescent's happiness**			0.50		0.50
Fair, happy or very happy	554 (97)	448 (81)		206 (46)	
Unhappy or very unhappy	18 (3)	16 (89)		6 (37)	
**Conflicts with adolescents**			0.90		< 0.01
Yes	235 (41)	191 (81)		104 (54)	
No	337 (59)	273 (81)		108 (40)	
**Adolescent aware of his/her HIV status**			< 0.01		0.10
Yes	501 (88)	419 (84)		198 (47)	
No or unsure	71 (12)	45 (63)		14 (31)	
**Age at ART initiation (years)**			0.40		0.06
0–6	180 (63)	144 (80)		75 (52)	
7–12	321 (56)	258 (80)		115 (44)	
>12	55 (10)	48 (87)		16 (33)	
**Adherence to treatment**			0.70		0.60
Good or very good	519 (91)	421 (81)		196 (47)	
Less than good	53 (9)	43 (81)		16 (37)	
**BMI** > **18.5**			0.40		0.08
Yes	206 (36)	163 (79)		66 (40)	
No	366 (64)	301 (82)		146 (48)	
**CD4 cell count (cell/mm** ^3^ **)**			0.40		0.07
>500	386 (67)	318 (82)		154 (48)	
≤ 500	186 (33)	146 (78)		58 (40)	
**Viral load (copies/mL)**			0.90		0.70
≤ 50	467 (82)	378 (80)		174 (46)	
>50	105 (18)	86 (82)		38 (44)	
**Characteristics of the caregivers**					
**Gender**			0.07		0.20
Female	445 (78)	354 (80)		167 (47)	
Male	127 (22)	110 (87)		45 (41)	
Age, Median, [IQR]	50 (41, 60)	52 (42, 61)	< 0.01	52 (42, 61)	0.50
**Relationship with the adolescent**			0.01		0.30
Parents	77 (13)	53 (69)		23 (43)	
Grandparents or siblings	217 (38)	184 (85)		86 (47)	
Aunt or uncle	138 (24)	117 (85)		46 (39)	
Other relatives	140 (245)	110 (78)		57 (52)	
**Caregiver's financial situation**			< 0.01		0.20
Good or very good	363 (64)	279 (77)		120 (43)	
Less than good	209 (36)	185 (88)		92 (50)	
**Caregiver own's health perception**			0.60		0.50
Good or very good	528 (92)	427 (79)		193 (45)	
Less than good	44 (8)	37 (84)		19 (51)	
**Member of a support group**			< 0.01		< 0.01
Yes	239 (42)	208 (87)		119 (57)	
No	333 (58)	256 (77)		93 (36)	

^*^Chi-square or student's test (significance is met when p < 0.05). Comparison of the adolescents whose HIV status is known in the community with the overall sample of adolescents living in family setting.

^**^Chi-square or student's test (significance is met when p < 0.05). Comparison of the adolescents with stigma/discrimination experiences reported by their caregivers with the overall sample of adolescents whose HIV status is known in the community.

^***^At least one parent alive vs. both parents known to be dead.

### 3.2 Knowledge of the HIV status in the community

Overall, 81% of the adolescents had their HIV status known in the community (Table 1). Disclosure was significantly more frequent in the North and North-East (*p* < 0.01), when the adolescents were orphan from both parents (*p* < 0.01), when they were aware of their own HIV status (*p* < 0.01), when the caregivers were older (*p* < 0.01), when their financial situation was not good (*p* < 0.01) and when the caregivers were members of an HIV support group (*p* < 0.01).

### 3.3 Report of stigmatization/discrimination among adolescents whose HIV status is known in the community

A total of 212 (46%) caregivers reported at least one occurrence of stigma/discrimination experienced by their child/adolescent (Table 1). Stigma/discrimination was significantly more frequent among those living in the Northeast region (*p* < 0.01), among those perceived by the caregiver to have an intellectual disability (*p* < 0.01), among those in conflicts with their caregiver (*p* < 0.01), and among those whose caregiver was member of a support group (*p* < 0.01). There was no statistical association with the orphan status, but there was a trend toward more frequent stigma/discrimination among adolescents who started ART before age 7 years (*p* = 0.06), those who had a BMI < 18.5 (*p* = 0.08), and those who had CD4 count >500 cells/mm^3^ (*p* = 0.07).

### 3.4 Context and types of stigmatization/discrimination experiences

Stigma/discrimination had primarily occurred at school in 64% of reports (from classmates, teachers or staff), from friends in 59% of the cases, and from family members in 10% of the cases (Table 2). Stigmatization in hospital was reported in < 1% of the cases. Twenty three percent of the caregivers reported repeated stigma/discrimination in the same context, and 24% in multiple contexts (Table 2).

**Table 2:** Prevalence, context and types of stigma and discrimination experiences, as documented by the caregivers.

**Stigma/discrimination experience**	***N* (%)**
Adolescents whose HIV status is known in the community	464
Any experience of stigma/discrimination	212 (46)
Repeated stigma/discrimination experiences	108 (23)
Diverse stigma/discrimination experiences	111 (24)
Circumstances of stigma/discrimination^*^(***N*** = **212)**	
At school	136 (64)
From friends	125 (59)
From people in the village	91 (43)
From family	22 (10)
At the hospital	5 (0.02)
Type of stigma/discrimination^*^(***N*** = **212)**	
Bullying/moral harassment	130 (61)
Social isolation	90 (42)
Behavioral discrimination	40 (19)
Public disclosure	16 (7)

^*^Adolescents could have experienced several types of stigma/discriminations, hence a total percentage above 100%.

### 3.5 Multivariable analysis

After adjustment for age and gender, the following covariates were included in the multivariable analysis: region, awareness of HIV status, perception of adolescent's intellectual abilities, conflicts with caregivers, age at ART initiation, BMI, CD4 count and caregivers' being membership of support groups. Factors independently associated with an experience of stigma/discrimination are presented in Table 3. The risk of being stigmatized, as perceived by the caregivers, was nearly three times higher in the northeast region (OR: 2.93, 95%CI: 1.36–6.45) and when having a low intellectual ability (OR: 3.35, 95%CI: 1.66–7.10). It was twice as high in case of conflicts with caregivers (OR: 1.81, 95%CI: 1.17–2.79), and when caregivers were members of a support group (OR: 2.28, 95%CI: 1.48–3.53). In contrast, having a BMI >18.5 was associated with a lower risk of stigma/discrimination (OR: 0.61, 95%CI: 0.37–0.98).

**Table 3:** Factors associated with stigma or discrimination after adjustment on age and gender.

**Factors**	**Any experience of stigma/discrimination**				**Diverse stigma/discrimination**				**Repeated stigma/discrimination**			
	**Univariable**		**Multivariable**		**Univariable**		**Multivariable**		**Univariable**		**Multivariable**	
	**OR (95%CI)**	***p***	**aOR (95%CI)**	***p***	**OR (95%CI)**	***p***	**aOR (95%CI)**	***p***	**OR (95%CI)**	***p***	**aOR (95%CI)**	***p***
**Age**		>0.99		0.15		0.90		0.99		0.48		0.90
12–13	Ref		Ref		Ref		Ref		Ref		Ref	
14–15	1.02 (0.67–1.57)		1.51(0.91–2.53)		0.94 (0.57–1.54)		0.99 (0.58–1.70)		1.05 (0.63–1.75)		1.28 (0.74–2.22)	
16–19	1.00 (0.61–1.65)		1.73(0.92–3.27)		1.14 (0.64–2.06)		0.98 (0.51–1.84)		1.57 (0.90–2.72)		2.13 (1.14–3.95)	
**Gender**		0.72		0.74		0.89		0.24		0.87		0.39
Female	Ref		Ref		Ref		Ref		Ref		Ref	
Male	1.07 (0.74–1.55)		0.93 (0.59–1.45)		0.97 (0.63–1.49)		0.75 (0.45–1.22)		0.93 (0.60–1.44)		0.80 (0.49–1.31)	
**Region**		0.07		0.03		0.02		0.06		0.01		0.01
Center	Ref		Ref		Ref		Ref		Ref		Ref	
North	1.38 (0.84–2.28)		1.46 (0.84–2.56)		1.07 (0.60–1.99)		1.21 (0.65–2.34)		0.80 (0.46–1.45)		0.75 (0.41–1.39)	
North–East	2.64 (1.31–5.40)		2.93 (1.36–6.45)		2.67 (1.25–5.78)		2.70 (1.21–6.13)		2.17 (1.04–4.58)		2.25 (1.04–4.94)	
South	1.68 (0.49–5.81)		3.31 (0.77–15.5)		1.94 (0.47–6.97)		2.29 (0.52–8.89)		0.63 (0.09–2.65)		0.54 (0.08–2.46)	
**School delay**		0.38				0.16				0.19		
No	Ref				Ref				Ref			
Yes	1.25 (0.76–2.04)				1.48 (0.85–2.53)				1.46 (0.83–2.50)			
**Orphan**		0.30				0.72				0.79		
No	Ref				Ref				Ref			
Yes	0.82 (0.57–1.19)				0.93 (0.60–1.42)				0.94 (0.61–1.45)			
**Adolescent aware of his/her status**		0.03		0.15		< 0.01		0.01		0.35		
No	Ref		Ref		Ref		Ref		Ref			
Yes	1.98 (1.04–3.95)		1.79 (0.81–4.12)		3.52 (1.38–11.9)		3.55 (1.30–12.6)		1.45 (0.69–3.44)			
**Perception of adolescent's health**		0.54				0.24				0.87		
Less than good	Ref				Ref				Ref			
Good or very good	0.87 (0.56–1.36)				0.74 (0.45–1.23)				1.04 (0.62–1.79)			
**Perception of adolescent's happiness**		0.50				0.50				0.87		
Fair, happy or very happy	Ref				Ref				Ref			
Unhappy or very unhappy	0.70 (0.24–1.93)				1.47 (0.45–4.13)				1.10 (0.30–3.24)			
**Perception of adolescent's intellectual ability**		< 0.01		< 0.01		< 0.01		< 0.01		< 0.01		< 0.01
Normal	Ref		Ref		Ref		Ref		Ref		Ref	
Low	3.10 (1.69–5.91)		3.35 (1.66–7.10)		2.45 (1.32–4.45)		2.99 (1.52–5.88)		2.40 (1.29–4.39)		2.92 (1.49–5.66)	
**Conflicts with adolescents**		< 0.01		< 0.01		0.01		0.07		0.15		0.53
No	Ref		Ref		Ref		Ref		Ref		Ref	
Yes	1.83 (1.26–2.66)		1.81 (1.17–2.79)		1.73 (1.12–2.66)		1.54 (0.96–2.49)		1.38 (0.89–2.13)		1.17 (0.72–1.89)	
**Age at ART initiation (years)**		0.07		0.06		0.47				0.68		
0–6	Ref		Ref		Ref				Ref			
7–19	0.69 (0.46–1.02)		0.64 (0.40–1.02)		0.76 (0.48–1.20)				0.91 (0.57–1.45)			
**Adherence to treatment**		0.30					0.94			0.79		
Less than good	Ref				Ref				Ref			
Good or very good	1.43 (0.72–2.92)				0.97 (0.46–2.24)				1.11 (0.52–2.69)			
**BMI** > **18.5**		0.09		0.04		0.92				0.98		
No	Ref		Ref		Ref				Ref			
Yes	0.71 (0.48–1.05)		0.61 (0.37–0.98)		0.94 (0.60–1.47)				0.99 (0.63–1.55)			
**CD4 cell count (cells/mm** ^3^ **)**		0.08		0.06		0.97				0.87		
≤ 500	Ref		Ref		Ref				Ref			
>500	1.43 (0.96–2.14)		1.57 (0.98–2.55)		0.98 (0.62–1.56)				1.04 (0.66–1.67)			
**Viral load**		0.74				0.03		0.07		0.50		
≥50	Ref				Ref		Ref		Ref			
< 50	0.92 (0.57–1.48)				1.78 (1.05–2.96)		1.63 (0.89–2.93)		1.20 (0.69–2.05)			
**Caregiver member of a support group**		< 0.01		< 0.01		< 0.01		< 0.01		< 0.01		< 0.01
No	Ref		Ref		Ref		Ref		Ref		Ref	
Yes	2.34 (1.61–3.42)		2.28 (1.48–3.53)		2.08 (1.35–3.21)		1.88 (1.18–3.04)		2.25 (1.46–3.51)		1.99 (1.24–3.23)	

OR, odds ratios; aOR, adjusted 95%CI, 95% confidence intervals.

p-value significant when < 0.15 in the univariable analysis; < 0.05 in the multivariable analysis.

Sensitivity analyses were conducted to assess the factors that remained associated with “repeated stigma/discrimination” and with “diverse stigma/discrimination” are shown in Table 3. Awareness of the adolescent's own HIV status (OR: 3.55, 95%CI: 1.30–12.6) was also associated with experience of “diverse stigma/discrimination.”

### 3.6 Qualitative analysis: experiences of stigma or social discrimination

All caregivers who reported experiences of stigma/discrimination toward their adolescents (N = 212) provided comments. In addition, 5 caregivers provided comments related to the fear of discrimination without having experienced it, and finally 9 caregivers reported discrimination from siblings (a category not considered separately in the questionnaire).

Inductive analysis of the verbatims identified four main themes of stigma: bullying or moral harassment was reported in 61% of cases, social isolation in 42% of cases, behavioral discrimination in 19% of cases, and public disclosure in 7% of cases (Table 2).

#### 3.6.1 Bullying or moral harassment

School was the main setting for bullying, where children/adolescents were mistreated by administrative staff teachers or friends. One of the most common forms of bullying was calling children “Pen AIDS,” i.e. “you have AIDS.” Such bullying sometimes led to temporary or permanent voluntary withdrawal from school. For three children who didn't know their HIV status, this was a way of finding out they were HIV-positive. Sometimes the bullying was also related to the health or HIV status of the parents.

Grand-father: “The child said that her friends often bullied her, “Pen AIDS,” so that she refused to go to school for one year.”
Aunt: “When the child was young, his friends always teased him that his parents got HIV and died from AIDS and that it will be the same for him. He came to me and asked what is AIDS?”

Bullying also occurred when children showed visible physical symptoms.

Aunt: “My niece could not go to school because the director said the other parents don't want a child who has skin wounds on her body.

#### 3.6.2 Social isolation

Another major issue raised by caregivers was the social isolation resulting from the children's HIV status. This isolation occurred at school, in the community and even within the family. The main reason given was the fear of infection.

Grand-mother: “The teacher didn't pay attention when my grand-daughter was asking permission to go out of the classroom in order to take her medication. Also when she was sick at school, she had to manage to call home on her own”
Mother: “The school director refused to enroll my son, because in that year, his own child was attending the school and he feared that they may be close”
Mother: “In kinder-garden, nobody dare to sit near my daughter”

In the community, parents of other children or neighbors also tended to forbid their children from playing with children who were known to be living with HIV.

Grand-mother: “My grand-daughter was always told that as having AIDS she was not allowed to play with other children at school and in the community”

Finally, in the family context, some comments were made about discrimination due to fear of contamination.

Aunt: “Some cousins were disgusted and feared that my niece might transmit the disease to other children. They warned her not to get close to other children”

#### 3.6.3 Behavioral discrimination

Another common discrimination behavior was to avoid sharing the same food or water with children living with HIV, whether at school, in the children's home or in any other place where the children might be invited.

Grand-mother: “My grand-daughter went to other people's house and ask them to drink water, but they refused”

Beyond not sharing food or water, some children were unable to access services or help because of their status.

Grand-mother: “Between age 8 and 9 years, when my grand-son was going for a haircut, the barber refused to do”

#### 3.6.4 Public disclosure

Some caregivers commented on how the child's HIV status was disclosed to the community.

Father: “I was angry at the teacher because she (the teacher) told the students that my child had HIV infection in front of the whole classroom”
Aunt: “The neighbor said that my niece had AIDS, and asked why she wasn't dying already. We both felt angry”

## 4 Discussion

Our study showed that, from the perspective of their caregivers, almost half of adolescents living with perinatal HIV had experienced stigma or discrimination. This occurred mainly at school, more often in the north-east region, was more likely to affect those perceived as having an intellectual disability, those in conflict with their caregivers, those with a BMI < 18.5, and those whose caregivers were members of support groups. It was not significantly associated with other factors such as gender, age, orphan status, health status including virological or immunological status, self-awareness of HIV status, or adherence to ART.

In our study, 19% of caregivers reported that their child's HIV status was not known to the community. In the Northern Thailand study of adolescents living with perinatal HIV followed in Chiang Mai University hospital, about 40% of caregivers reported keeping their adolescent's HIV status secret, a percentage twice as high as in our study (31). The lack of disclosure in the community is likely related to anticipated stigma (35), as caregivers or adolescents may have chosen not to disclose their status (36). However, they are not always in a position to choose disclosure, as for example, the HIV status of orphans is generally known in the community, with parental deaths suspected to be HIV-related. In addition, people living with HIV who are in the most precarious situation are more likely to apply to the dedicated financial support from the government. As registration is often not confidential, the HIV status is then known in the community. It is difficult to compare our findings with those of other studies conducted in Thailand, as the populations and approaches used to study stigma/discrimination against adolescents are different. Similar to our study, a qualitative study conducted in Bangkok among 33 adolescents and young adults 15–24 years (more than half of whom were infected perinatally) indicates that the educational context was the main setting in which they encountered stigma/discrimination (37). In contrast with the study in Chiang Mai University hospital, where almost half of the caregivers had discrimination attitudes toward their adolescents (31), caregivers in our study reported relatively few (10%) experiences of stigma/discrimination in the family context. It is possible that this frequency is underestimated, as specific questions about discriminatory attitudes in the family were not asked, and that caregivers may not consider these attitudes to be discriminatory. In neighbor Cambodia, similar frequencies of HIV stigma/discrimination were measured, ranging from 32.0 (38) to 43.2% (39). Finally, experiences of stigma/discrimination in the health care setting were almost never reported (< 1%). This contrasts with the situation in the United States, where a study of adolescents and young adults found that 38% had experienced HIV-related stigma/discrimination when accessing sexual health services, particularly women (40).

In our study, the most common expression of stigma/discrimination against children was teasing or bullying, reported by about a quarter of all caregivers. In the Chiang Mai study, 23% of caregivers reported that their adolescents had been teased and 11% bullied at school (31), a similar percentage to our findings. In our study, social isolation was mentioned by almost a fifth of all caregivers and was reinforced in case of physical symptoms. The word “rangkiat,” meaning “disgust,” was often used by caregivers to express how HIV-infected people were perceived by others.

We found that the prevalence of stigma/discrimination was highest in the Northeast region. This is consistent with the results of successive surveys on stigmatizing attitudes toward people living with HIV among the Thai adult population (29, 30), and highlights the need to prioritize this region for interventions.

Our study also shows a strong association between stigma/discrimination and lower intellectual ability among adolescents. In a previous analysis, it was found that adolescents who had experienced stigmatization at school were almost twice as likely to have a disrupted academic trajectory (10). However, it is difficult to distinguish between HIV-related stigma and stigma associated with mental disability. Negative consequences of stigma on mental health have also been documented (41–44). Finally, we found that reports of stigma/discrimination were more frequent when caregivers were members of an HIV support group. Through their participation in these groups, caregivers are better informed about HIV and may be more sensitive to stigma and discrimination. Also, the association between stigma/discrimination, and conflicts with caregivers might indicate resentment toward parents who transmitted the virus perinatally, or toward caregivers who may view them as a burden (45).

We found no difference in the experience of stigma/discrimination according to gender. This is in contrast to findings in adults, where women were more likely to experience stigma/discrimination than men (27). Similarly, unlike in adults, HIV-related stigma/discrimination was not associated with poor adherence (15). In fact, during childhood, adherence depends primarily on the caregivers who provide ART and directly observe its intake (46, 47). There was also no association between stigma/discrimination and the viral load level or CD4 count. It should be noted that the virological and immunological response to ART was very good in both groups. As encouraging results in medical settings are found both in our population and other populations (48), stigma remains a factor that can facilitate the spread of HIV (49). The transition to adulthood remains complex and should be managed in a multidisciplinary way, including psychosocial support (50).

Thailand has developed several interventions to prevent HIV stigmatization based on three building blocks: policy, measurement and implementation (50). Among the results, reducing stigma and discrimination was mentioned as a goal in the National AIDS Strategic Plan (2014–2016) and was high on Thailand priority agenda. In addition, surveillance data was obtained from the general population, healthcare professionals and key-populations. Moreover, participatory interventions and training took place in healthcare facilities. Finally, the United Nations has created a mobile telephone application enabling people to contact HIV services in Bangkok to report stigma or discrimination events and monitor them (51).

Our findings may not be representative of the situation in the country, as most adolescents were recruited from the northern region and were living in family settings. Adolescents living in orphanages may have a different experience of stigma and discrimination. However, our study was conducted on a relatively large sample of adolescents living with perinatal HIV, recruited from 20 hospitals of different sizes across Thailand.

The study was carried out more than 10 years ago, and it is known that, in adults, stigma and discrimination have decreased as a consequence of the widespread use of ART (52). However, the level of stigma/discrimination in perinatally-infected children and adolescents were, to our knowledge, not measured in the last years. In the 5th and 6th Thai National Health Examination Surveys, which measured stigmatizing attitudes toward people living with HIV among adults in the general population, one-fourth and one-fifth, respectively, believed that HIV-infected children should be separate from other children at school (29, 30). However, in the Thailand National Strategic Plan 2017–2030, documented interventions do not mention schools or other settings involving this specific population. Therefore, although the data was collected relatively long ago, describing stigma/discrimination experiences of adolescents born with HIV remains essential to highlighting their specific needs, which must be addressed in Thailand and other contexts, particularly in Africa.

The 46% prevalence of stigma/discrimination obtained from interviews with caregivers is likely to be an underestimate, as children/adolescents do not always report their experiences to their caregivers, and direct testimonies from adolescents were not available. Also, given the frequent change in caregivers due to parental separation or death, the caregiver at the time of the interview may not be aware of past experiences of child stigma or discrimination. However, our approach of interviewing caregivers rather than the adolescents themselves, while reducing the number of reports of stigma/discrimination, respected the fact that some adolescents had not been informed of their HIV status and prevented them from recalling painful events in their lives. Nonetheless, the fact that information was collected from the caregivers might lead to an information bias. This is indicated by the higher odds of perceived stigma when caregivers were members of a support group. The age of the children/adolescents at the time of HIV disclosure in the community and when their stigma/discrimination experiences occurred, as well as internalized self-stigma were not available. A specific Thai internalized HIV-stigma scale for adults was developed in 2023 (53). However, the mixed methods used, with a life-history approach in which the adolescent's life was reconstructed by the caregiver, alongside a qualitative questioning, enabled us to obtain detailed information about stigma/discrimination experiences in childhood and adolescence.

Thanks to the successful implementation of PMTCT, the number of adolescents living with perinatal HIV is decreasing significantly (54) and may no longer be considered a key population. However, as they reach adulthood, they may face the consequences of past discrimination in terms of access to university studies or certain jobs, and may face discrimination at work, in the community or in their romantic life (55). We are currently conducting a follow-up study, TEEWA-2, in which the same young people will be asked directly about their current experiences of stigma/discrimination, as young adults (56).

In its National Strategy to End AIDS 2017–2030, Thailand has committed to reducing HIV-related discrimination by 90% (57), and has outlined measures to promote awareness of HIV, human rights, and sexuality in healthcare settings, the media, and the workplace. However, the strategy does not directly address children and adolescents born with HIV, or include specific interventions in schools or settings involving this population. While Thailand can be considered a success story in its fight against HIV/AIDS, in terms of prevention of sexual transmission, PMTCT, scaling up ART and combating stigma/discrimination among adults (58), our study highlights the vulnerability of children and adolescents for whom community-based and school-based interventions should be targeted.

## Data Availability

The raw data supporting this study will be made available upon reasonable request, contingent upon approval by the authors.
